# Reconsidering tolerability of cancer treatments: opportunities to focus on the patient

**DOI:** 10.1007/s00520-021-06700-0

**Published:** 2022-01-11

**Authors:** John Devin Peipert, Mary Lou Smith, On Behalf Of the EVOLV Study Team

**Affiliations:** 1 Department of Medical Social Sciences, Northwestern University Feinberg School of Medicine, 625 Michigan Ave, 21st Floor, Chicago, IL 60611 USA; 2 Research Advocacy Network, Chicago, IL USA

**Keywords:** Cancer, Patient, Tolerability

## Definitions of treatment tolerability

There has been a significant movement toward increasing the patient’s voice in assessment of tolerability of cancer treatments [1]. Traditional assessment of tolerability has relied on clinician-rated toxicities, using the National Cancer Institute’s Common Terminology Criteria for Adverse Events (CTCAE). A recent white paper published by the Friends of Cancer Research advanced the ability to assess tolerability from the patient’s perspective: “The tolerability of a medical product is the degree to which symptomatic and non-symptomatic adverse events associated with the product’s administration affect the ability or desire of the patient to adhere to the dose or intensity of therapy. A complete understanding of tolerability should include direct measurement from the patient on how they are feeling and functioning while on treatment.”[2] This definition improves upon previous considerations of tolerability. Most importantly, it shifts focus to how a patient feels or functions in response to their treatments. It also implies that tolerability is assessed via patient report. We agree with and support this new, patient-oriented definition of tolerability, but we believe there are important opportunities to expand it. The Friends’ definition focuses exclusively on experience with treatment, which is critical but captures only what happens while the patient is on treatment. In addition, there is a need to capture what the patient is bringing to the table even before starting treatment that can indicate their tolerability. Our focus in this perspective is to reveal the importance of the patient disposition in addition to the patient experience in capturing treatment tolerability.

## Tolerability is related to characteristics of individual patients

A patient-focused approach to tolerability should focus on patient characteristics that directly or indirectly convey the patient’s ability or desire to stay on treatment. We argue that this entails two broad categories of indicators. First, as noted by the Friends’ paper, there is pertinent experience data, which includes patient-reported side effect burden, patient-reported symptoms, and patient-reported outcomes like physical function. These factors excel at capturing the patient’s interpretation of symptom or side effect severity and frequency, and less often, interference in daily activities and roles. Of course, these indicators contribute value to capturing tolerability from the patient’s perspective, but they only tell part of the tolerability story in that they focus on results after the treatment has been taken and not on the factors that potentially predispose patients to vary in their responses to treatment. Such predisposing indicators include patient preferences around the treatment (e.g., treatment administration schedule), attitudes (e.g., values the treatment), stressful life events (e.g., loss of job, change of residence), history with treatment, sensitivities to specific side effects, and potentially several other individual factors. These dispositional factors could potentially be captured as global willingness to stay on treatment even when there are barriers or complications. These characteristics may vary widely from individual patient to individual patient. Due to the inter-patient variability in these characteristics, there will be substantial variation between individual patients in their ability to tolerate a specific treatment. In other words, different patients will react differently to the same toxicities associated with a given treatment due to these characteristics.

## Recommendations for measurement of tolerability

As we have argued above, assessing treatment tolerability requires capturing key indicators of both the patient experience and patient disposition aspects of tolerability. Table 1 shows examples of indicators for each. There are already several good options for capturing the patient experience of tolerability. The patient-reported outcomes version of the National Cancer Institute’s Common Terminology Criteria for Adverse events (PRO-CTCAE) makes available the ability to assess the frequency, severity, and level of interference associated with 78 of the most common symptomatic AE’s experienced by patients in cancer trials [3]. This system represents a very flexible approach capturing patient experience data relevant to tolerability, especially if the composite scoring options are used to summarize across the frequency, severity, and interference for an AE domain (e.g., fatigue) [4]. In addition, the Functional Assessment of Chronic Illness Therapy (FACIT) measurement system makes available an item library covering many relevant cancer side effects in terms of single items and multi-item scales [5]. Within the FACIT system is a single item (GP5) from the Functional Assessment of Cancer Therapy – General (FACT-G) measure, “I am bothered by side effects of treatment,” which captures side effect burden on a global level [6, 7]. Because adverse effects are so varied, GP5 responses can facilitate comparisons across treatments or cancer types more easily than assessments that focus on specific types of side effects selected to suit the circumstance at hand. Recent guidance from the US Food and Drug Administration (FDA) has recognized both the PRO-CTCAE and GP5 as promising approaches for capturing patient experience with cancer treatment. [8] These measures represent an important aspect of tolerability, but they are only part of the total picture of tolerability.

**Table 1:** Key indicators of the patient experience and disposition aspects of treatment tolerability

Patient experience	Patient disposition
Overall side effect burden	Overall willingness to stay on treatment while enduring side effects
Specific adverse events	Preferences for treatment
Functional ability	Attitudes toward treatment

In addition to patient experience, we suggest assessment of patients’ disposition toward staying on treatment. To do so, we must tap the patient’s preferences and attitudes about treatment, including willingness to stay on treatment even while enduring side effects. This captures the essence of tolerability, at least as viewed from the patient perspective. This might include questions such as: “Do you feel you can stay on your treatment even if you have side effects?” A combination of patient experience and patient disposition indicators of tolerability will likely comprise the most comprehensive assessment approach, though more research will be required to determine the optimal combination.

Finally, we call attention to the unit of consideration, if not the unit of measure, in patient-reported tolerability assessment. There is reference in the Friends’ definition to determining the level of tolerability of a cancer treatment. First, patient-focused tolerability assessment focuses on understanding what the individual patient’s characteristics and experiences reveal about their capacity to tolerate different treatments. Such assessment is actionable at the individual patient level because it can be used to facilitate shared treatment decision-making. This point of view generates new hypotheses about whether or not particular patient characteristics are facilitators or barriers to tolerability treatments. In addition, as clinical trials aim to evaluate the treatment, individual patient reports on tolerability can be aggregated (e.g., averaged) and compared at the treatment level. The recommendations for patient-reported tolerability described here will facilitate both of these needs.

## Data Availability

N/A.

## Code availability

N/A.
