# Correction to: Reconsidering tolerability of cancer treatments: opportunities to focus on the patient

**DOI:** 10.1007/s00520-022-07276-z

**Published:** 2022-07-12

**Authors:** John Devin Peipert, Mary Lou Smith, On Behalf Of the EVOLV Study Team

**Affiliations:** 1 Department of Medical Social Sciences, Northwestern University Feinberg School of Medicine, 625 Michigan Ave, 21st Floor, Chicago, IL 60611 USA; 2 Research Advocacy Network, Chicago, IL USA

**Correction to: Support Care Cancer (2022) 30:3661–3663**

**https://doi.org/10.1007/s00520-021-06700-0**

The article "Reconsidering tolerability of cancer treatments: opportunities to focus on the patient", written by Peipert, J.D., Smith, M.L., and On Behalf Of the EVOLV Study Team, was originally published Online First without Open Access. After publication in volume 5, page 3661–3663 the author decided to opt for Open Choice and to make the article an Open Access publication. Therefore, the copyright of the article has been changed to © The Author(s) 2022 and the article is forthwith distributed under the terms of the Creative Commons Attribution 4.0 International License, which permits use, sharing, adaptation, distribution and reproduction in any medium or format, as long as you give appropriate credit to the original author(s) and the source, provide a link to the Creative Commons licence, and indicate if changes were made. The images or other third party material in this article are included in the article’s Creative Commons licence, unless indicated otherwise in a credit line to the material. If material is not included in the article’s Creative Commons licence and your intended use is not permitted by statutory regulation or exceeds the permitted use, you will need to obtain permission directly from the copyright holder. To view a copy of this licence, visit http://creativecommons.org/licenses/by/4.0.

